# Unusual Colonic Iron Deposition Following Prophylactic Supplementation in an Infant: A Case Report

**DOI:** 10.1002/ccr3.72346

**Published:** 2026-06-01

**Authors:** Farid Imanzadeh, Mohammad Mahdi Heidari, Seyyed Ramin Madani, Fatemeh Mahjoub, Somayyeh Jameie

**Affiliations:** ^1^ Pediatric Gastroenterology, Hepatology and Nutrition Research Center, Research Institute for Children's Health Shahid Beheshti University of Medical Sciences Tehran Iran; ^2^ Department of Pediatrics, School of Medicine Iran University of Medical Sciences Tehran Iran; ^3^ Aliasghar Clinical Research Development Center, Department of Pediatrics, School of Medicine Iran University of Medical Sciences Tehran Iran; ^4^ Department of Pediatrics, Faculty of Medicine Kashan University of Medical Sciences Kashan Iran; ^5^ Department of Pathology Tehran University of Medical Sciences Tehran Iran; ^6^ Faculty of Medicine, Tehran Medical Sciences Islamic Azad University Tehran Iran

**Keywords:** colon, gastroenterology, iron deposition, pediatrics

## Abstract

Iron plays a vital role in physiological functions, and its deposition is typically limited to reticuloendothelial tissues in the presence of excess levels. We report a rare case of colonic iron deposition in a 13‐month‐old child without iron overload or toxicity. Despite a normal serum iron profile and administration of only prophylactic iron supplementation, histological examination revealed marked iron accumulation in the lamina propria of the colon. This unusual presentation emphasizes the need for awareness of possible iron‐related mucosal changes even in the absence of systemic overload, particularly in children with gastrointestinal or immunologic comorbidities.

## Key Clinical Message

Colonic iron deposition can occur in infants receiving only routine prophylactic iron supplementation, even with normal serum iron levels. Clinicians should consider this rare finding in cases of unexplained gastrointestinal symptoms to avoid unnecessary invasive interventions.

## 1 Case Presentation

**FIGURE 1:**
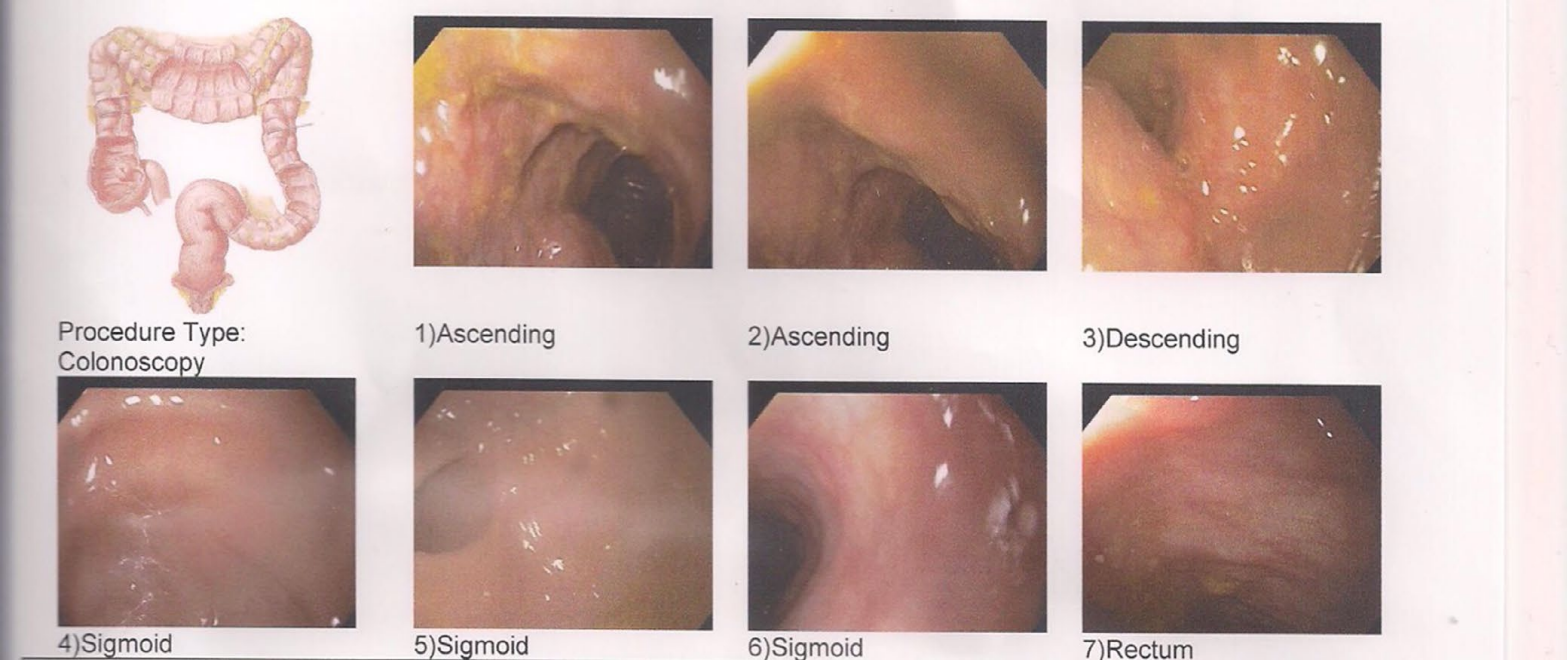
Nodularity was seen at the right colon.

A 13‐month‐old male infant was referred to a pediatrician due to inadequate weight gain beginning at 7 months of age. Initial evaluation included a skin prick test, which revealed sensitivities to cow's milk and peanuts. He was started on an elimination diet along with medications including ketotifen (Zaditen) and esomeprazole (Nexium). Despite the use of medication, his weight gain didn't improve. From 9 months of age, the patient began experiencing episodes of vomiting, diarrhea, and intermittent fever up to 39°C, occurring every 5 days and resolving spontaneously. Prick allergy test repeated within 6 months showing sensitivity to cow's milk, almond, walnut, and additives. Also, baclofen therapy was added to other medications. Due to the country's routine treatment, he was taken ferrous sulfate drop at a dose of 2 mg per kg per day from 6 months of age for prophylactic treatment of anemia. Laboratory evaluation showed a gradual increase in ESR, with normal serum iron, ferritin, and vitamin D levels. Immunoglobulin assay revealed low IgA. Due to the positive calprotectin in the stool, colonoscopy and colon biopsy were done for him. Nodularity was seen at the right colon (Figure 1).

Pathology report stated: Histopathology revealed mucosal irregularity, decreased goblet cell density, and numerous brown granules consistent with iron deposition within the lamina propria, confirmed by special staining. A prominent lymphoid aggregate was also observed. His weight was 8800 g and his height was 78 cm for his age. Following discontinuation of iron supplementation and introduction of an extensively hydrolyzed formula, the patient's clinical condition improved significantly, with resolution of symptoms and catch‐up growth. Over the following months, the patient demonstrated improved tolerance to feeding, reduced gastrointestinal symptoms, and a more stable weight trajectory. Close follow‐up by pediatric gastroenterology and immunology teams was maintained. At the latest follow‐up visit, the patient was clinically stable, with catch‐up growth observed and no recurrence of febrile episodes or gastrointestinal complaints.

## 2 Discussion

This case presents an uncommon finding of colonic iron deposition in an infant without systemic iron overload or excessive iron intake. Studies have shown that fractional iron absorption from breast milk is higher in unsupplemented infants compared to those receiving iron supplements, suggesting a regulatory mechanism affected by supplementation [1]. According to Domell, the study of iron absorption was directly associated with dietary iron intake but not with indexes of iron status, including plasma ferritin [1]. Iron is primarily stored intracellularly as ferritin, which serves as a buffer against both iron deficiency and overload [2]. Several studies have documented upper gastrointestinal mucosal injury in adults receiving therapeutic doses of oral iron supplements [3, 4]. Proton pump inhibitors such as esomeprazole, which our patient had been receiving for gastroesophageal reflux symptoms, are frequently used in infants and have been shown to be effective and generally well‐tolerated [5]. The type and formulation of iron supplements have been shown to significantly influence their potential for mucosal toxicity [6]. The liver is the primary site for iron storage, and hepatic iron overload is the most common finding in cases of systemic hemosiderosis [7]. Increased deposition of iron within the liver may contribute to liver disease via the production of reactive oxygen species [8]. Excess iron can enter the body by two distinct pathways: increased gastrointestinal absorption or intravenous blood transfusion. Genetic hemochromatosis, erythropoietin hemochromatosis, and Bantu siderosis are examples of conditions that result from long‐term increased absorption of iron through the bowel. In such conditions characterized by iron absorption, iron deposits into tissue parenchyma, which affect hepatocyte initially, and as the disease progresses deposition can also occur in the pancreas and heart [9]. The iron deposition in the heart, liver, and endocrine system without increasing iron storage in the reticuloendothelial system occurs in hemochromatosis of the newborn. Iron deposits in the mitochondrial system cause symptoms in Friedrich's ataxia. However, iron deposition within the gastrointestinal tract is uncommon and is usually associated with systemic conditions such as hereditary hemochromatosis [10]. Intravascular hemolysis, not chronic transfusion, causes renal hemosiderosis in sickle cell disease [11]. Iron deposition without evident erosive mucosal injury is also seen in a small proportion (14%) of biopsies in Abraham's article [12]. It is reported three cases of iron deposition in the duodenum in Dr. Mahjoub's article [6]. Wilson Kwong̓ s report demonstrated hemosiderosis in multiple areas of the gastrointestinal tract including the stomach, duodenum, and jejunum [7]. Iron poisoning happens in doses higher than 20 mg per kg per day, and may create symptoms such as abdominal pain, diarrhea, gastrointestinal bleeding, and causes mucosal damage in the intestines and also damage the heart and liver. Serum levels of iron in these patients are high [13]. To our knowledge, this is the first reported case of colonic iron deposition in an infant receiving only routine prophylactic iron supplementation. Our patient used a prophylactic dose of iron and serum iron level was normal. The underlying mechanism of colonic iron accumulation in the absence of systemic overload remains unclear and warrants further investigation.

## 3 Conclusion

Iron deposition in the colon without iron overload is a rare and poorly understood finding in pediatric patients. Clinicians should consider this entity in cases of unexplained gastrointestinal symptoms, particularly when routine iron supplementation is used. Further studies are warranted to explore underlying mechanisms and to assess whether additional screening is necessary in such scenarios.

## Author Contributions

**Farid Imanzadeh:** conceptualization, supervision. **Mohammad Mahdi Heidari:** investigation, writing – original draft. **Seyyed Ramin Madani:** data curation, writing – original draft. **Fatemeh Mahjoub:** conceptualization, supervision. **Somayyeh Jameie:** writing – review and editing.

## Funding

This work was supported by Takeda Science Foundation.

## Ethics Statement

The study received ethics approval from the Ethics Committee of the Kashan University of Medical Sciences (IR.KAUMS.REC.1402.023). Written informed consent was obtained from the patient's legal guardian (father) for publication of this case report and any accompanying images.

## Data Availability

The data that support the findings of this study are available from the corresponding author upon reasonable request.
